# Data analytics to evaluate the impact of infectious disease on economy: Case study of COVID-19 pandemic

**DOI:** 10.1016/j.patter.2021.100315

**Published:** 2021-07-27

**Authors:** Meleik Hyman, Calvin Mark, Ahmed Imteaj, Hamed Ghiaie, Shabnam Rezapour, Arif M. Sadri, M. Hadi Amini

**Affiliations:** 1 Sustainability, Optimization, and Learning for InterDependent Networks Laboratory (solid lab), Knight Foundation School of Computing and Information Sciences, Florida International University, Miami, FL 33199, USA; 2 Economics and Public Policy at ESCP Business School, 75011 Paris, France; 3 Enterprise and Logistics Engineering, Florida International University, Miami, FL 33174, USA; 4 Moss School of Construction, Infrastructure & Sustainability, Florida International University, Miami, FL 33174, USA

**Keywords:** coronavirus, COVID-19, dataset, machine learning, economic impact, stock market

## Abstract

SARS-CoV-2 (COVID-19) is a new strain of coronavirus that is regarded as a respiratory disease and is transmittable among humans. At present, the disease has caused a pandemic, and COVID-19 cases are ballooning out of control. The impact of such turbulent situations can be controlled by tracking the patterns of infected and death cases through accurate prediction and by taking precautions accordingly. We collected worldwide COVID-19 case information and successfully predicted infected victims and possible death cases around the world and in the United States. In addition, we analyzed some leading stock market shares and successfully forecast their trends. We also scrutinized the share market price by proper reasoning and considered the state of affairs of COVID-19, including geographical dispersity. We publicly release our developed dashboard that presents statistical data of COVID-19 cases, shows predicted results, and reveals the impact of COVID-19 on leading companies and different countries' job markets.

## Highlights

• We developed a time-series model that can effectively predict COVID-19 cases
• We reveal the impact of COVID-19 on the world's economy and unemployment rate
• We publicly release our developed COVID-19 dashboard

## The bigger picture

SARS-CoV-2 (COVID-19) is a new strain of coronavirus that is regarded as a respiratory disease, and the number of COVID-19 cases is ballooning out of control. The impact of COVID-19 can be controlled by tracking the patterns of infected and death cases through accurate prediction. We developed a COVID-19 dashboard by collecting worldwide COVID-19 case information and successfully predicted COVID-19 infected victims and possible death cases. In addition, we analyzed some leading stock market shares and successfully forecast their trends using multiple linear regression. In addition, we scrutinized the share market price by proper reasoning and considered the state of affairs of COVID-19, including geographical dispersity. We publish a dashboard that demonstrates the effectiveness of our proposed learning model showing predicted results for future COVID-19 cases and reveals the impact of COVID-19 severity on leading companies and different countries' job markets.

## Teaser

We developed a model that can predict COVID-19 infected and death cases. Our developed model almost accurately predicts total COVID-19 cases worldwide (accuracy 99.3%), total COVID-19 cases in the United States (accuracy 99.1%), total death cases worldwide (accuracy 99.27%), and total death cases in the United States (accuracy 96.9%). In addition, we analyzed some leading stock market shares and successfully forecast their trends. We considered Dow Jones, S&P 500, and NASDAQ market prices and obtained an accuracy of 96.8%, 96.8%, and 93.5%, respectively.

## Introduction

COVID-19 has become a widely growing pandemic that is threatening the health of the earth's global population, and its emergence is having a severe impact on the global economy. In 2020, unemployment rates skyrocketed, and just in the United States tens of millions of non-essential businesses have been shut down.^1^ Therefore, the first 6–12 months of 2021 are crucial as the worldwide economy needs to rebound as soon as possible. Leaders, economists, and other officials need to make informed decisions following the most effective way to reopen the economy. In addition, it is vital for policy makers to make full economic analyses such that the consequences can be understood ahead of time, as reopening too soon could create a second spike in COVID-19 cases and has the possibility of making the situation worse than the current spike. In this regard, policy makers need comprehensive analyses to predict risk. To make decisions regarding the reopening of different sectors, we need an early forecast of the probable time when the COVID-19 severity is going to be reduced, and the economy will rebuild. After that, proper mitigation steps can be implemented such that trade-offs between public health and the economy can be quantitatively considered.

There are many factors of COVID-19 that remain unknown. As the pandemic matures and a sufficient amount of data is available, more clarity can be gained regarding how to combat this pandemic. In addition, as the fatality rate of COVID-19 is unknown, it would require a large-scale survey to detect specific antibodies to COVID-19. It is estimated that the COVID-19 case fatality rate is 0.3%–1%, as opposed to that of influenza A, which is about 0.1%. Second, it is unknown whether infectiousness starts before the onset of symptoms. Data suggest that the incubation period of COVID-19 is roughly 5–6 days. Further studies^2^, ^3^, ^4^ indicate the possibility of viremia being high enough that transmission could occur within 1–2 days of contact, which brings into question how contagious COVID-19 actually is. Third, it is uncertain what the magnitude of asymptomatic COVID-19 cases is. Data suggest that roughly 80% of those who come in contact with COVID-19 have mild symptoms or are asymptomatic, 14% of cases are severe in nature, and 6% of cases are of a critical nature.^5^ This suggests that symptom-based control is not likely to be sufficient unless cases can remain only lightly infectious. Finally, the duration of the infectious period for COVID-19 is unknown. Based on a few clinical virological studies, it is estimated that the infectious period is about 10 days or more after the incubation period.^6^ Evidence suggests that a second, more intense spike can occur if social distancing protocols are broken too soon, and that may cause an inevitable negative impact on financial markets.^7^^,^^8^ From the beginning of the outbreak, the financial markets have tried to respond to regular developments considering the global pandemic. We can see effects in the labor supply, which are attributed to three areas: mortality due to infection, morbidity due to infection, and morbidity arising from caregiving for affected family members. Upon announcement of the pandemic, it was expected to incite panic and, therefore, sent shocks into the equity risk premium of economic sectors. Consequently, the production of supplies, as well as the demand for consumption, would be affected. To avoid such situations, governments must implement measures to combat the economic downturn. This should be done by keeping the viral spread as low as possible, by extending social distancing guidelines that ensure a low mortality rate, and making comprehensive plans for economic reopenings that balance safety. In other words, governments should not open fully, but in phases. As COVID-19 emerged before any major mitigation efforts were identified and implemented, we could see small chains of transmission expanding into a significant spread in countries such as Italy, Iran, South Korea, and Japan. On the other hand, the effects of social distancing and quarantining in China have shown they are a viable method of case reduction. If we consider a base reproduction number that defines the mean number of secondary cases generated by a primary one, then, in the case of a population that is largely susceptible, around 60% of the global population could be infected.^9^ In such a case, epidemiologists would face difficulties in helping policy makers take objective decisions toward mitigation, minimizing mortality, and avoiding an epidemic peak that overwhelms health care services. This will also aid in keeping the economy within manageable levels while attending to the epidemic until a vaccine can be successfully developed. Further, legislation needs to be provided to furnish both short-term and long-term policy responses. In the short term, the central government's role is to employ central banks and treasuries to make sure that disrupted economies can continue to function while the outbreak continues. In the long term COVID-19 can create a global, large-scale disruption. It is vital that individual governments invest sufficiently into their own health care systems while backing the public health systems of less developed countries, where it is more likely for diseases to originate and spread due to lack of infrastructure and which can become hotbeds of infection. As diseases are not isolated or selective, they can spread and kill people from any socioeconomic group, making any disease epicenter a global risk. The potential costs can be cut through cooperative investment globally, particularly in public health and development that includes investments in Third World sanitation infrastructures, investments in global public health, and lower interest rates by central banks. A few papers have proposed to analyze the COVID-19 impact on human lives,^10^ environment,^11^ unemployment and suicide,^12^ economy,^13^ health care,^14^ education,^15^ food behavior,^16^ and human mobility.^17^ These paper explained how COVID-19 is extensively influencing human lives. Although some papers^18^, ^19^, ^20^ proposed learning models for forecasting COVID-19 cases, their results are not live and they did not consider the COVID-19 effect on economy.

In this paper, we mainly aim to develop a learning model that can leverage data analytics to evaluate COVID-19 severity across different countries and its impact on the global economy. Our main contributions can be listed as follows:

• We collected COVID-19 data from reliable sources to visualize the past and present COVID-19 state with detailed information.
• We developed a time-series model using the concept of the Holt-Winters (HW) seasonal method that can effectively predict COVID-19 cases (e.g., world cases, daily COVID-19 cases, total death cases, etc.).
• Applying the multiple linear regression (MLR) method, we revealed the unavoidable impact of COVID-19 on the world's economy and showed how a nation's unemployment rate is directly connected with the COVID-19 strike.
• We publicly released our developed dashboard that aims to display all our simulations with necessary details.

### Preliminaries of the problem and existing models

The effects of COVID-19 can be observed in different sectors, and the prolonged existence of this disease can cause a huge crisis in global health and the economy. Consider a scenario in which COVID-19 causes a long-lasting supply disruption. Then, a persistent drop in labor productivity can be expected. The fall in productivity will translate into lower aggregate demand. The central bank reacts by cutting the policy rate, but not enough to prevent unemployment from rising. The result is a drop in employment, which, according to models, might require central banks to give further monetary stimulus. It is inconclusive what effect this will have on inflation as many other factors need to be considered. Therefore, as COVID-19 triggers a shock in the supply-demand doom loop, it also places the economy in a danger zone in which expectations can affect employment and productivity growth. In this case, central banks will be unable to counteract the associated drop in demand. As a result, employment and economic activity drop. Firms react by cutting investment, which negatively affects productivity growth. Initial pessimistic expectations of weak growth thus become self-fulfilling. This self-fulfilling feedback loop can take place only if the fundamentals of the economy are weak. In addition, there is a greater chance of overall spending when the shocks are concentrated in particular sectors that have high demand due to the response to the pandemic. The fact that some goods are no longer available makes it less attractive to spend overall. The increase in prices of goods in the affected sectors discourages consumption as goods become more expensive. The best policy combines containment policies that will shut down a particular sector, e.g., sector 1. Consider a social insurance policy that provides compensation to the workers in sector 1, a monetary policy that hits the natural rate, and the government introduces a social insurance policy that reallocates income from another sector (sector 2). If reallocation is geared to equalize the workers’ after-transfer incomes, this policy will make it easier to achieve demand stabilization, making it easier and more cost effective for a government to pursue public health objectives. Positive demand may indeed overreact to the supply shock and lead to a demand-deficient recession by loosening monetary policy, providing abundant social insurance, and lower taxes while holding spending constant can aid in increasing economic output. Further, Fornaro and Wolf, McKibbin and Fernando, Loayza and Pennings, and Coibion et al.^21^, ^22^, ^23^, ^24^ discussed COVID-19 and its effect on macroeconomic policy. As the COVID-19 pandemic continues to spread across the world, it is apparent that the virus will have a negative effect on the global economy. We can see the virus causing a negative supply shock in the global economy by forcing factories to shut down for extended periods of time, disrupting the global supply chain. Glover et al.^25^ developed a model coupling economics and health care to understand the economic impact of COVID-19. They considered individuals who were dissimilar in age, health status, and sector. They proposed to construct a model of multiple disease transmission sources from the level of economic activity. Their results showed that partially shuttered sectors are most heavily affected by COVID-19. To mitigate the spread of COVID-19, many countries are shutting down non-essential sectors of the economy. The effects of this will differ per demographic. Consider older individuals who may have more to gain from quarantine orders, as this demographic is at higher risk of harm from COVID-19. On the other hand, younger individuals, who make up most of the workers across many sectors, may be more affected financially as many are unable to work.^26^

The SIR (susceptible-infected-removed) model^27^ can be effective in showing the progression of the COVID-19 pandemic. Stock^28^ proposed a more powerful SIR epidemiological model that helps us to understand social distancing and economic containment policies by evaluating their impact on the economy. He emphasizes the importance of collecting COVID-19 testing data following proper guidelines. Considerable gaps in data collected from cases may, in turn, play into the effectiveness of policy response to COVID-19. Stock expressed his model regarding the transmission rate of infection propagation and asymptomatic rate (infected people who do not have any symptoms or mild symptoms) following Bayes law. He estimated the asymptomatic rate of COVID-19 by considering a random sample or a fraction of the overall population for testing. The economic consequences and policy design can be greatly influenced by the asymptomatic rate or undetected infected population. We can assume that most people mix homogeneously, and as a result, the undetected population is as infectious as the symptomatic. Ridenhour et al.^29^ mentioned that the spread of an epidemic within a population can be divided into three categories: susceptible to infection (*S*), infected, and no longer contagious (either by death or recovery) (*R*). The transmission rate *R*_*t*_ can be determined by measures of social distancing. It is estimated that the health system will be overwhelmed when the fraction of infected people is over 1% of the population.^30^ From Equations 1, 2, 3, 4, and 5, we express the evolution of the fraction of population being infected over time:

(Equation 1) $$\frac{ds}{dt}=-\beta_{t}\frac{S}{N}I,$$

(Equation 2) $$\frac{dE}{dt}=\beta_{t}\frac{S}{N}I-\sigma E,$$

(Equation 3) $$\frac{dI}{dt}=\sigma E-\gamma I,$$

(Equation 4) $$\frac{dR}{dt}=\gamma I,$$

(Equation 5) $$\beta_{t}=R_{t}\gamma ,$$

where γ, σ, and $\beta_{t}$ denote the rate per day, incubation period, and rate of contact spread, respectively.

Fernandes, Ozili and Arun, Lenzen et al., Carlsson-Szlezak et al., Song and Zhou, and Legese Feyisa^31^, ^32^, ^33^, ^34^, ^35^, ^36^ discuss the effects of COVID-19 on the global economy under different lengths of shutdowns. The World Trade Organization expects global trade to fall as every sector of global trade will be affected. The COVID-19 pandemic is unlike any crisis that existed before, where global health, supply, and demand are simultaneously in a state of crisis. Furthermore, having already exhausted their power before the pandemic, central banks have offered only zero or negative interest rates, preventing central banks from providing significant aid. For each country, the model uses GDP decomposed into its different economic sectors. It is assumed that service-oriented sectors will be more affected during the crisis months than agriculture or industry. The model assumes that countries with a larger tourism sector will be more severely affected than countries that are more industrial focused. McKibbin et al.^22^ estimate the economic cost of the COVID-19 outbreak and how the disease might evolve under seven different scenarios. Of seven scenarios, the first three assume that epidemiological events occur only in China and it is isolated from other countries. Still, the economic impact on China spills over to other dependent countries through financial markets, capital flows, and trade. In the next three scenarios, they consider that the epidemiological shocks spread worldwide to different degrees of severity. The first six scenarios are expected to remain for a temporary time period. In the last scenario, they consider a mild pandemic to occur every year for an indefinite future. A proper indicator of the COVID-19 impact on the economy is the continuous fluctuation of the financial market indices. We figured out that the COVID-19 pandemic and its continuous evolution throughout the world is highly volatile and uncertain, making it challenging for the decision-maker to undertake pertinent macroeconomic policies. In addition, we can observe a deficiency in demand for most of the daily products that caused supply shocks due to the low substitutability across sectors, incomplete markets, and liquidity-constrained consumers.^37^, ^38^, ^39^ These factors are contributors to the possibility of Keynesian supply shocks. Therefore, proper policy, fiscal tools, and monetary policies need to be addressed to overcome the shocks. In the theory of a Keynesian supply shock, a shock would trigger aggregate demand changes that would be larger than the shock itself. A simple perspective on the effects of COVID-19 casts the issue as one of aggregate supply versus aggregate demand, whether the shock to one side is greater than the other. When workers lose their income, their spending reduces, causing a contraction in demand.

As the COVID-19 pandemic continues to evolve, little is known about what long-term macroeconomic effects it can have. Currently, most attention is focused on the short-term impact. Jordà et al., McKee and Stuckler, Malliet et al., Nicola et al., Fuchs-Schündeln et al., and Fana et al.^40^, ^41^, ^42^, ^43^, ^44^, ^45^ studied the long-term economic consequences of a pandemic. They focused on the previous major pandemics in which over 100,000 people died and analyzed the after-effects of those pandemics, particularly on global macroeconomics. They collected data from the 14th century to their time of investigations, and prepared a continuous time series to measure annual frequency of the corresponding economic performance in different regions, cities, and countries. They figured out that some of the pandemics’ impacts persisted for around 40 years, and their results allow for understanding historic macroeconomic responses to pandemic events. The natural response rate can be defined as:^40^

(Equation 6) $$\hat{\tau }\left(h\right)=E\left(r_{t+h}^{\text{∗}}-r_{t}^{\text{∗}}|P_{t}=1;{\text{Ω}}_{t})-E(r_{t+h}^{\text{∗}}-r_{t}^{\text{∗}}|P_{t}=0;{\text{Ω}}_{t}\right),$$

where, $r_{t+h}^{\text{∗}}-r_{t}^{\text{∗}}$ refers to the change in the natural rate from the year the pandemic ends to a future time *h*. *P*_*t*_ can be set to 1 considering a pandemic ending in *t*. ${\text{Ω}}_{t}$ refers to information available at *t*. Therefore, $\hat{\tau }\left(h\right)$ of Equation 6 can be estimated using local projections. By measuring the natural rate of interest and deviations of different economic statistic benchmarks, these responses specify that the impacts of pandemics are generally continued for sustained periods. From the analysis of past pandemics over a span of decades, it is expected to see low investment opportunities with the possibility of heightened desire to save or rebuild depleted wealth. If COVID-19 follows a similar trend, maintaining low interest rates for decades, welcome fiscal space can be provided for governments to alleviate the pandemic consequences.^40^

Krueger et al.^46^ argue that internal conveyances in private consumption behavior can be helpful during an epidemic once temporary lockdowns are lifted. They suggest that well-operating labor markets, private assets, and social insurance policies can mitigate the economic decline and cost of life due to COVID-19 spread. For instance, Sweden does not impose any hard restrictions regarding lockdown on its citizens and allows its citizens to make their own decisions. Interestingly, the disease spread in Sweden is in line with most of the other European countries that imposed hard restrictions regarding lockdown, but the decline in economic growth is considerably mitigated. Krueger et al.^46^ showed that heterogeneous sectors have lower infection rate compared with homogeneous sectors. During an outbreak, households substitute required goods of high-infected sectors with those of low-infected sectors while following a stable consumption path. In consequence, the infection rate and numbers of deaths would be remarkably mitigated. In addition, effective social planning for COVID-19 can isolate the infected and susceptible people from the healthy and recovered people. Proper isolation of the infected and susceptible people can stop the further spread of the disease and can stabilize the world economy.

As global recession seems inevitable, lawmakers can implement polices that can affect the degree of preventative measures taken within a population. To prevent further spread of COVID-19, lockdowns may be induced as a form of mitigation. Consequently, this can have negative social and economic effects. Some partial/complete lockdowns may lead to a decrease in consumption and production interruptions, disrupting the global supply chain. China has reportedly passed its peak in the number of COVID-19 cases and has begun to reopen its economy. China's average small and medium enterprise (SME) is projected to be at an average of a 60% work rate with an estimated 9 million unemployed workers (according to the Economist Intelligence Unit). It is projected that western countries are 1.5–2.5 months behind China in terms of the outbreak. Therefore, it is crucial to begin reopening the economy. Although the length of this crisis is unknown, it is important to reemphasize the proper reopening of the economy to prevent a global resurgence of COVID-19 cases, which can further decimate a crippled economy.

As there is currently no guarantee of completely abolishing the existence of the COVID-19 virus, the question remains as to what the appropriate monetary and fiscal policies will be in the future. Here, we consider three possible scenarios:

• First, to handle the downfall of global demand, a monetary stimulus may be helpful to alleviate the COVID-19 impact on global employment.
• Second, we can observe a supply-demand doom loop in COVID-19 situations that could be exacerbated by the supply disruption.
• Third, we might face economic stagnation due to the high unemployment and low growth episodes driven by the COVID-19 pandemic, and to avoid such stagnation, aggressive and sensible fiscal policies need to be created by policy makers.

In the next section, we discuss our developed forecasting model for COVID-19 spread by considering the time-series data and constructing a visual representation of our results that can help us to understand the COVID-19 spread in terms of daily infected cases, total infected cases, death rate, and economic and unemployment status of different countries and also may help us to reveal the impacts of infectious disease (e.g., COVID-19) on the economy.

### System description

The choice of an appropriate forecasting procedure depends on a variety of considerations such as forecasting objective, number of available observations, and number of variables to be predicted. The HW methodology^47^ is an effective and widely used forecasting strategy that can generalize simple exponential smoothing to cope with the trend and seasonal variations. The HW method assumes that the local depersonalized mean level may be modified by an additive trend term, and it considers an additive error term of constant variance. The user must decide whether to use the additive or multiplicative seasonal model or a non-seasonal model. Another strategy proposed by Groff led to a different result regarding the accuracy of the Box-Jenkins (BJ) method.^48^ In time-series analysis, the BJ method figures out the best fit for the time-series model by applying an autoregressive integrated moving average on the past time-series values. However, the results of Goodwin^48^ might be invalidated based on the fact that the BJ identification process was not used for each series.^48^ Instead of that, simple projection methods are often preferred to the BJ procedure for a variety of practical considerations, such as cost, available expertise, and the fact that the BJ procedure requires at least 50 observations to have a good chance of success. Of course, it is sometimes argued that the HW method is optimal for a special case of the autoregressive integrated moving average class of models, the implication being that one might as well use the more general BJ procedure.

Indeed, what is remarkable about the Newbold-Granger and Reid comparisons is that HW actually performs better than BJ for about one-third of the series. Finally, in defense of the HW procedure against the empirical results of the Newbold-Granger and Reid studies, it must be said that these comparisons were not altogether fair from one important aspect. While the results give important guidance on the relative accuracy of completely automatic methods, they say little about the comparison with the BJ procedure because, if one is thinking of using a complicated method like BJ, it would seem fairer to compare it with non-automatic versions of the simpler procedures. After all, it would be a miracle if the completely automatic HW procedure outperformed the BJ procedure on average, although, as we have seen, it does actually do this for about one-third of the series.

One interesting feature of the results reported by Newbold and Granger was that BJ sometimes gave much better forecasts than HW, whereas HW was rarely superior to BJ by more than a small margin. This was perhaps to be expected given that the BJ procedure allows a choice from a much wider class of models. By using series for which BJ gave much better forecasts than automatic HW, one would endeavor to answer two queries. First, do series of this type have any general features that would yield guidelines for indicating when BJ is likely to be preferable to automatic HW and, second, can the HW results be improved by using a non-automatic version of the method. Unfortunately, records no longer exist of the BJ models fitted in the Newbold-Granger comparison or of the mean-square forecast errors that resulted from using the BJ or automatic HW methods. The absence of detailed records on the Newbold-Granger comparison raises a general query about empirical studies, which are not available for reexamination. Admittedly, any improvements that we make on the automatic HW procedures will be improvements on our findings only, but there is good reason to suppose that similar improvements will be possible for the Newbold-Granger automatic results. To check on this, we reestimated the HW smoothing parameters by minimizing mean absolute one-step-ahead forecast errors, which are evaluated under experimental results. To construct a COVID-19 forecasting model, at first we need to prepare a time series. The time series holds the previous data of COVID-19 cases with respect to time. We need to ensure that we apply a sufficient and variant amount of data during model training so that the model becomes neither biased nor overfit. After collecting the necessary data, we need to conduct an exploratory analysis on the collected data. It is necessary to check the completeness of the collected data by identifying null or duplicated values, looking at the shape of the dataset, and determining how many unique values there are in each column. Once the preprocess is completed, the next objective is to find the best method for developing a model using statistical analysis and machine learning techniques. After the collected data are plotted, a pattern is generated that clearly shows the difference between number of COVID-19 cases during the weekend and during weekdays. We use the HW methodology,^47^ which is one of the most widely popular forecasting methods for time series. HW forecasting is a technique for modeling and predicting the behavior of a time series. The HW seasonal method comprises the forecast equation and three smoothing equations: one for the level, one for the trend, and one for the seasonal component, with corresponding smoothing parameters α, β, and γ. Such a strategy provides us with all the flexibility needed to get the right fit to our data. HW applies exponential smoothing to perform encoding on the values from the past and utilize those historical data to forecast values for the present and future. The generated model predicts values by evaluating the aggregated influences of the level, trend, and seasonal components.

For the HW exponential smoothing method, we first consider that the trend is added to the current level, while the seasonality is multiplicative. This is a common scenario of time-series data and for that, we consider the equation:

(Equation 7) $$F_{\left(a+b\right)}=\left(L_{a}+b*B_{a}\right)\text{∗}S_{\left(a+b-p\right)},$$

where $F_{\left(a+b\right)}$ represents the forecast at step *a* + *b*, $\left(L_{a}+b*B_{a}\right)$ indicates estimated level at step *a* + *b*, and $S_{\left(a+b-p\right)}$ denotes estimated seasonal variation for a period of length *p* at step *a* + *b*. We start from an arbitrary step *a* and forecast the *b* time steps out in the future. Our main focus is to estimate $L_{a}$, $B_{a}$, and $S_{a}$. At first, we estimate the trend $B_{a}$ at step *a*:

(Equation 8) $$B_{a}=\beta *\left[L_{a}-L_{\left(a-1\right)}\right]+\left(1-\beta \right)\text{∗}B_{\left(a-1\right)},$$

where B_*a*_ represents the estimated trend at step *a*, $\left[L_{a}-L_{\left(a-1\right)}\right]$ indicates the estimated rate of level change from step *a* − 1 to step *a*, and $B_{\left(a-1\right)}$ denotes estimated trend at step *a* − 1. Then, we estimate the level $L_{a}$ at time step *a* as follows:

(Equation 9) $$L_{a}=\alpha *\frac{T_{a}}{S_{\left(a-p\right)}}+\left(1-\alpha \right)\text{∗}\left[L_{\left(a-1\right)}+B_{\left(a-1\right)}\right],$$

where $L_{a}$ represents the estimated level at step *a*, $T_{a}$ indicates the time series value at step *a*, and $S_{\left(a-p\right)}$ represents the seasonal component at step *a* − *p*. For estimating the value of $L_{a}$, we also need to figure out the seasonal component S_(*a* − *p*)_ at step *a*, which can be estimated as follows:

(Equation 10) $$S_{a}=\gamma *\frac{T_{a}}{L_{a}}+\left(1-\gamma \right)\text{∗}S_{a-p},$$

where $S_{a}$ represents the estimated seasonal component at step *a* and $L_{a}$ indicates the level at step *a*.

Now, as we know the required equations for estimating level, trend, and seasonal component at time step *a*, we can perform the estimation of the forecast F_(*a* + *b*)_ at step (a+b) as follows:

(Equation 11) $$F_{\left(a+b\right)}=\left(L_{a}+b*B_{a}\right)\text{∗}S_{\left(a+b-p\right)},$$

where $\left(L_{a}+b*B_{a}\right)$ represents the estimated level at step *a* + *b* and $S_{\left(a+b-p\right)}$ indicates the estimated seasonal variation at step *a* + *b* for period of length *p*. So, in this way, we construct our time-series model to forecast COVID-19 cases. For checking our model performance, we compare the difference between actual COVID-19 cases and the predicted ones, and tune up our model as we obtain more available COVID-19 data. After our model is trained with a sufficient amount of COVID-19 data, the closeness of the actual line and the predicted line for different COVID-19 scenarios (e.g., world COVID-19 infected cases, daily COVID-19 death cases) is increased. We figure out that the HW seasonal method performs extremely well in predicting COVID-19 cases because it can handle a large number of complicated seasonal patterns by finding the average value of the given input and adding the slope and seasonality effects of those values. For instance, during prediction of worldwide COVID-19 cases, we consider 627038 as the label for COVID-19 cases that is generated from the given input, multiply the number by date, and add an exponential smoothing value (i.e., −2.77286 × 10^10^). In a similar way, worldwide daily cases, world deaths, US cases, US daily cases, unemployment, and GDP are predicted, with the same concepts having different labels and exponential smoothing. After that, we prepare a model for simulating the impact of COVID-19 on worldwide GDP and unemployment rate. We apply the MLR method^49^ to predict both GDP and unemployment rate for different countries. MLR is a statistical technique that takes the input of multiple explanatory or independent variables for forecasting the outcome of a response or dependent variable. The main aim of MLR is modeling linear relationships between explanatory and response variables. We consider the following equation for performing MLR to forecast GDP and unemployment rate:

(Equation 12) $$y_{n}=\delta_{0}+\delta_{1}x_{n1}+\delta_{2}x_{n2}+\ldots +\delta_{w}x_{np}+\varepsilon ,$$

where *y*_*n*_ is a dependent variable, *x*_*n*_ is an independent variable, *δ*_0_ is the y intercept, *δ*_*w*_ is the coefficient of the independent variable, and *ε* is the model residual. For forecasting GDP and unemployment rate, we consider location, frequency, and time period. We train our model using GDP and unemployment data of past years and carry out MLR to predict future effects due to COVID-19. We calculate the residual by comparing the actual curves with our predicted curves (for both GDP and unemployment rate) and tune up our models accordingly.

## Results and discussion

### Experimental results

Albert Allen Bartlett famously stated that the most significant shortcoming of the human race is our inability to understand the exponential function.^50^ As cases of COVID-19 began to rise, there became an alarming exponential trend with cases worldwide and, later on, within the United States. Understanding this, we aim to develop a model that would accurately forecast the number of cases in various world regions. We begin by collecting case data from sources such as the European Centre for Disease Prevention and Control,^51^ the World Health Organization,^52^ and worldometer.^53^ While looking for data sources, our main objective was to look into the accuracy of case reporting, how often the datasets were updated, and the feasibility of converting the data into a time series. Once we isolated these three sources, we narrowed down our choice to just one. The worldometer dataset fits all the criteria for our model.

#### Data collection

We collected COVID-19 data on confirmed cases and deaths from the European Centre for Disease Prevention and Control,^51^ worldometer,^53^ the US Centers for Disease Control,^54^ and the World Health Organization.^52^ Data included the total number of cases in over 100 countries, including the epicenter, mainland China, and countries with the most COVID-19 cases (e.g., United States, India, Brazil). After collecting the data, we organized them into a series or one-dimensional array with respect to time. The dates were used as an index and national cumulative total in each row. We collected the COVID-19 map data from Johns Hopkins University CSSE,^55^ and economic data are from Worldbank,^56^ Bureau of Economic Analysis,^57^ and Yahoo Finance.^58^

#### Data analytics and discussions

We used Python^59^ in a Jupyter Notebook to develop our model and imported necessary libraries such as ExponentialSmoothing, SimpleExpSmoothing, and Holt from statsmodels.tsa.api. We reformatted our data into a list object and then placed it into Python's Panda data frame,^60^ where each row was indexed according to the date the data were collected. Since data were collected daily, we decided that it would be optimal to set our model's frequency daily, which assisted in interpreting the model. For example, if any significant changes to our forecast's slope or a large jump in cases could be easily recognized, and if changes were needed, they could be performed in a timely manner. While fitting the data to our forecast model, we conducted goodness of fit by evaluating R squared to avoid overfitting and underfitting. Some of the duplicates involuntarily dampened the projection, and values of zero caused errors in our code. To resolve these issues, we removed data from 3 weeks earlier in the year and kept only days with consistent new cases reported. Such a strategy provided an optimal starting point for the model, which significantly improved our trend and seasonality values. When the models were running, we noticed that our forecasted values were larger than the actual values by about 5%. We resolved this issue by incorporating slope damping. We found that 92% was optimal for the best forecast through trial and error, reducing variance by 3%–4%. Once our model was optimized and run, we had an alpha above 0.917 for all models. Once our forecast models were completed, we started this process to compare COVID-19 cases and deaths against the economy. Our first goal was to understand what key indicators showed the characteristics of the economy and specific industries and then to gather the necessary data from a reputable source. Our research on the economy showed that the stock market was a great indicator of how well industries were performing.

Meanwhile, the GDP and unemployment rate show how well a country's economy and potential growth are doing quarterly. Taking this information into consideration, we were able to find a great collection of data from the World Bank and the stock market. We tried to figure out the correlation between the number of new cases of COVID-19 and the financial stability of some industries. Our main goal was to determine what industries were being hit the hardest by COVID-19 and their correlation with other interdependent networks. We laid out a plan to get an exact figure on which growing restrictions and social distancing guidelines hit industries the hardest. Our team decided the best way to show this relationship was through a correlation matrix of COVID-19 cases and deaths with the key indicator of the largest industries, such as gas, oil, transportation, manufacturing, etc. We began by placing the data collected into a Panda data frame. Then, Pearson's R correlation was applied to identify the relationships between the growing number of COVID-19 cases and various industries.

#### Results analysis

**Figure 1:**
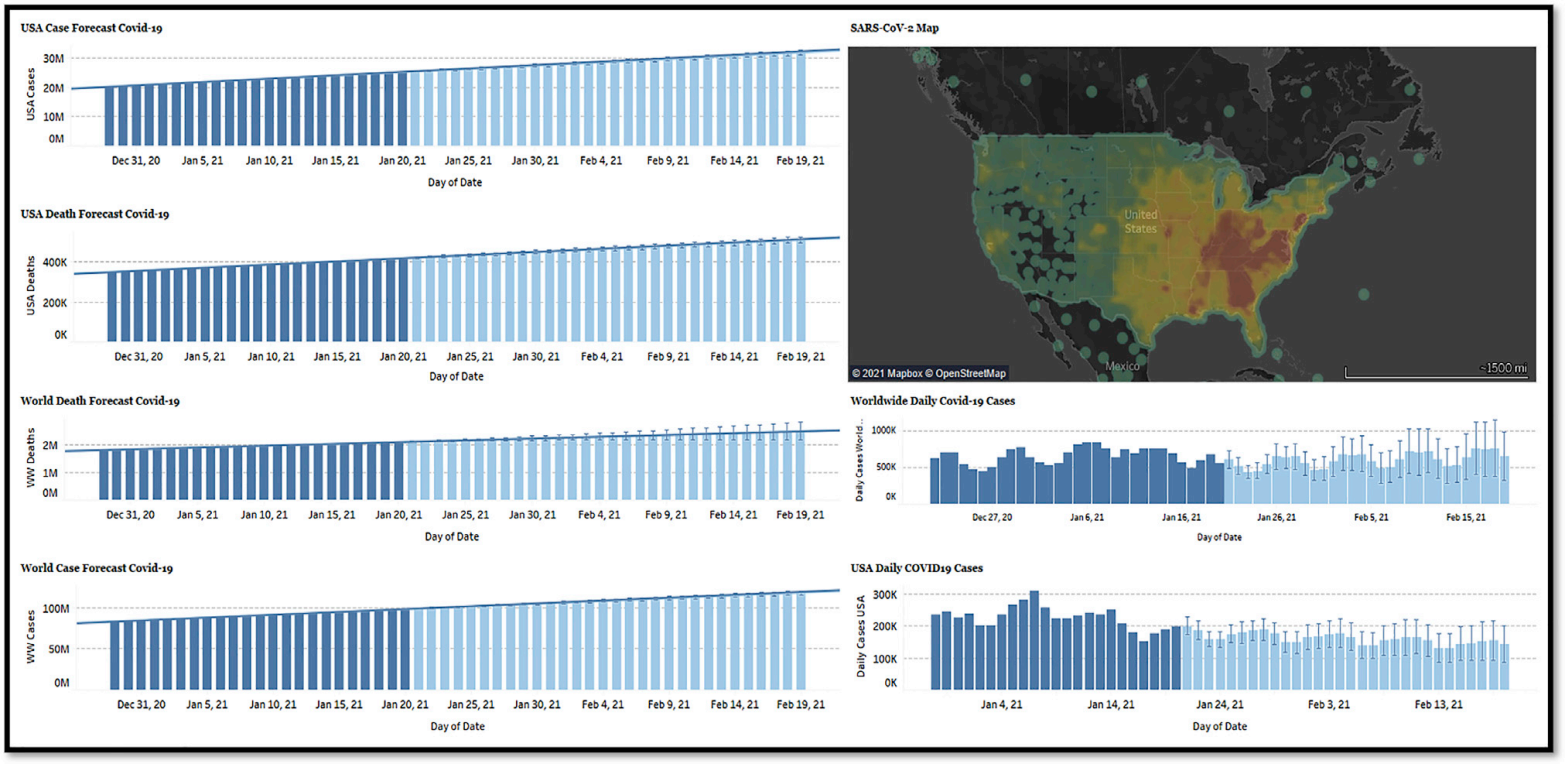
Our developed COVID-19 statistical forecasts and visualizations dashboard

**Figure 2:**
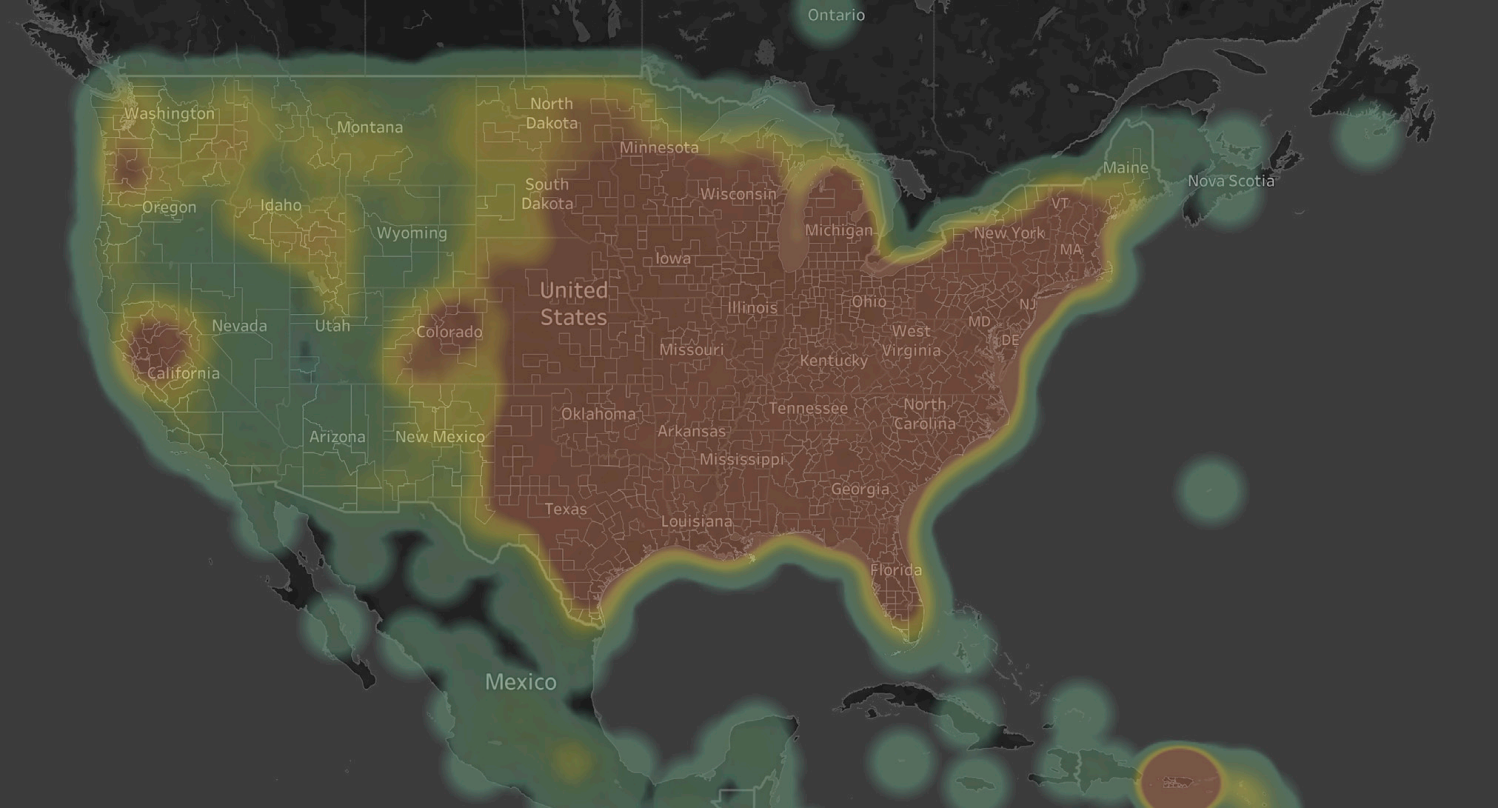
Visualization of COVID-19 severity on the US map

Our developed data analytics dashboard for COVID-19 prediction is publicly available (https://public.tableau.com/profile/solid.lab\#!/vizhome/DataAnalyticsforCOVID-19Prediction/Covid-19Dashboard), and we present the dashboard in Figure 1. The dashboard highlights all the predicted COVID-19 cases (e.g., daily cases, death cases) that we deployed. In Figure 2, we illustrate the COVID-19 severity on a map that allows users to observe COVID-19 severity in different regions.

**Figure 3:**
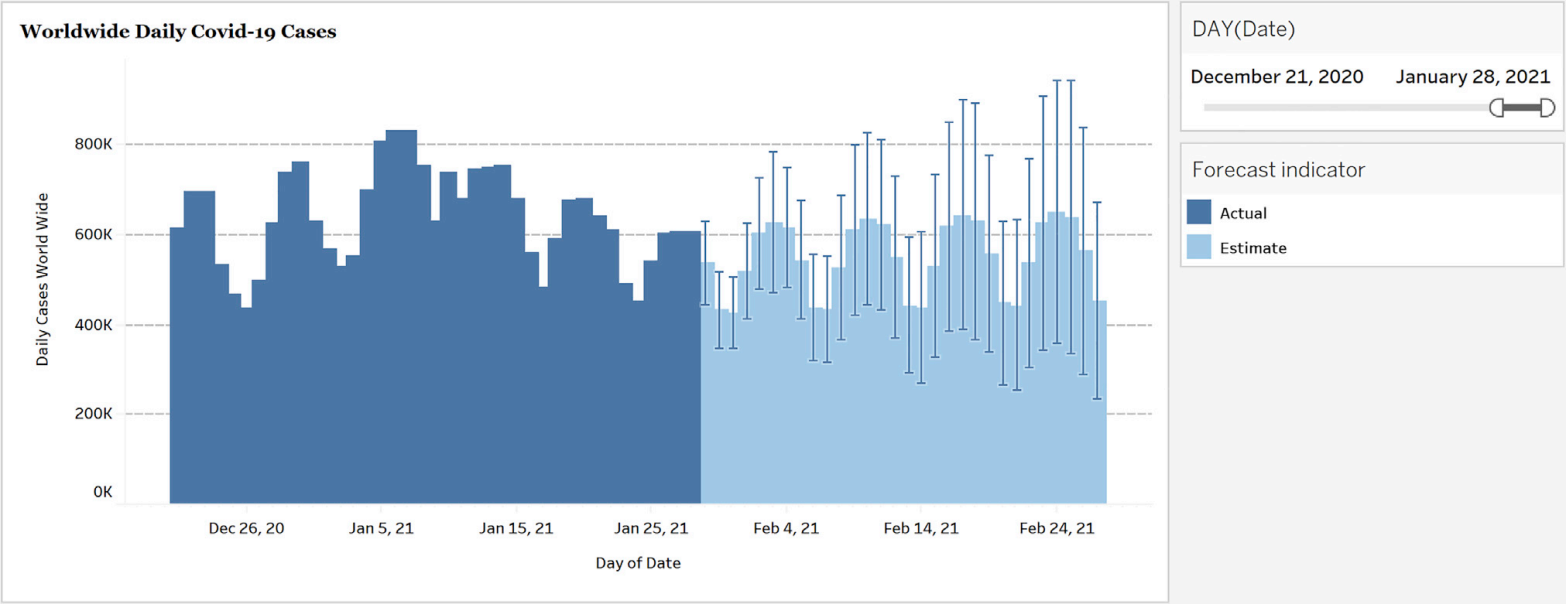
Forecasting daily COVID-19 infected cases around the world

**Figure 4:**
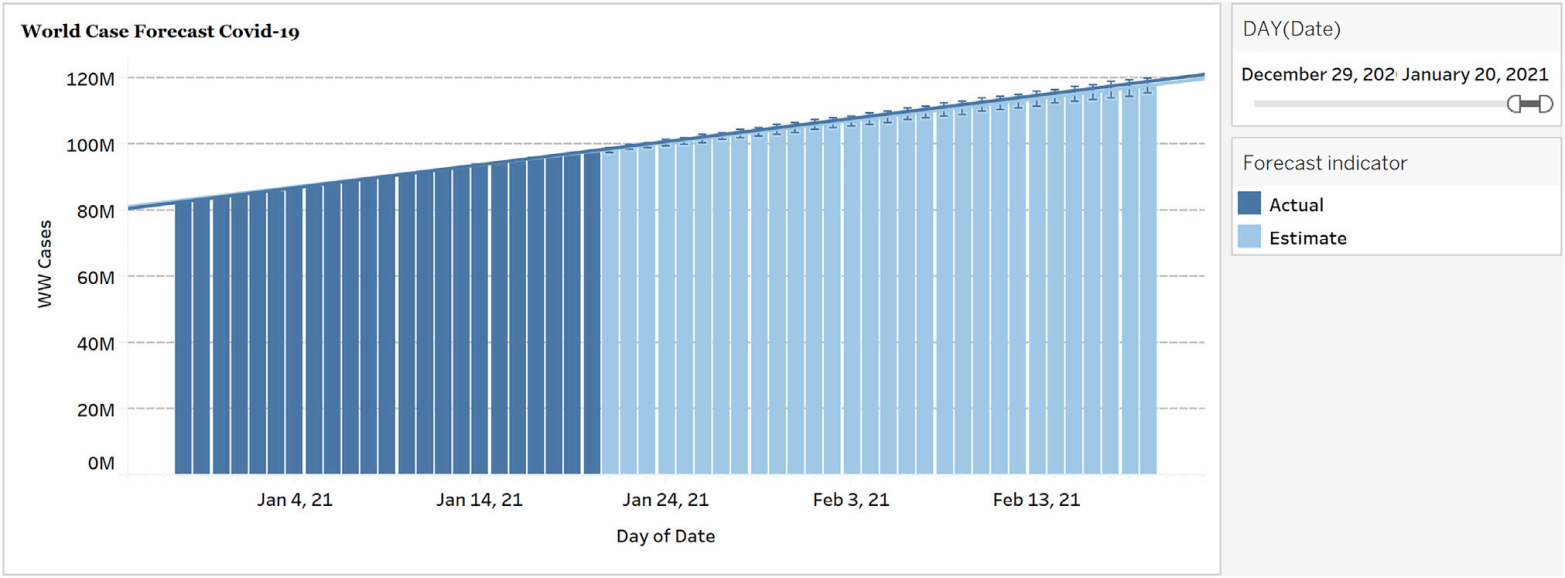
Forecasting total COVID-19 cases around the world

**Figure 5:**
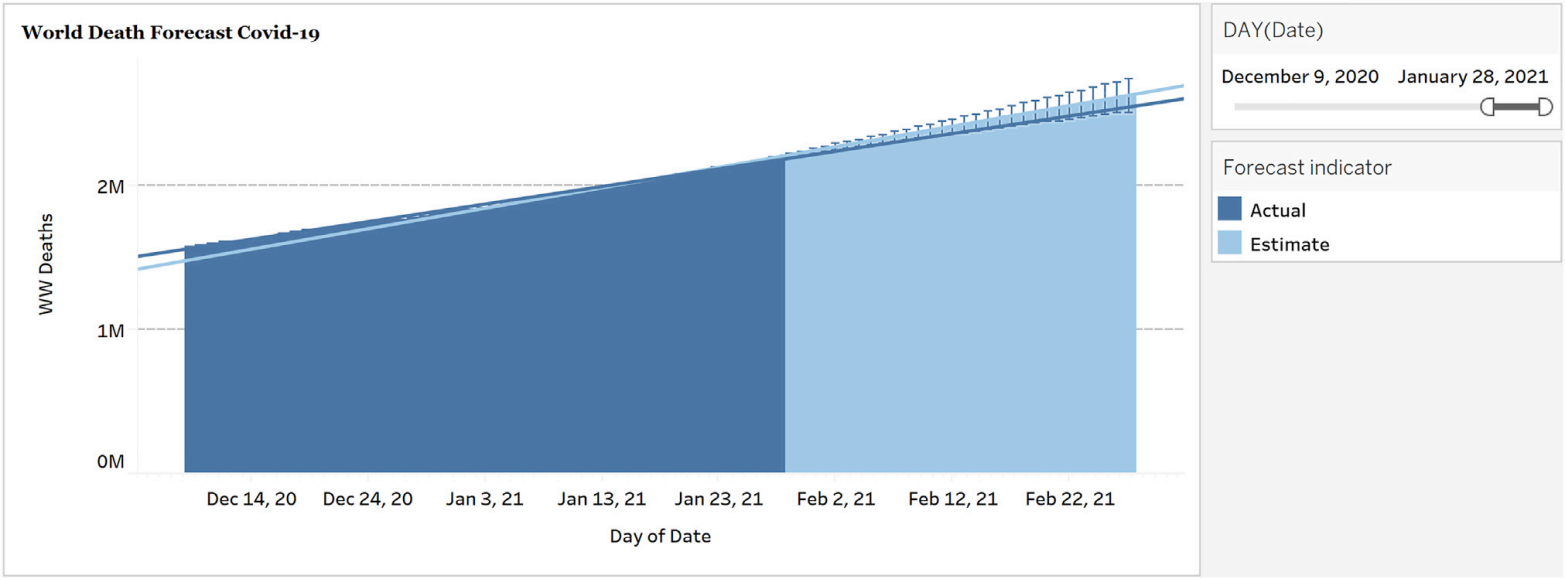
Forecasting COVID-19 death cases around the world

In Figure 3, we present worldwide daily COVID-19 cases and we can observe that COVID-19 daily cases follow a specific pattern. In particular, the daily COVID-19 cases during the weekends are lower than during the weekdays. After that, we present the worldwide total COVID-19 cases in Figure 4. We can observe that the cases are increasing linear and the actual and estimate forecast indicator lines are almost aligned. Figure 5 also shows a linear increment of the death cases due to COVID-19, and the closeness of the actual and the estimate forecasting lines demonstrates the reliability of our constructed model.

**Figure 6:**
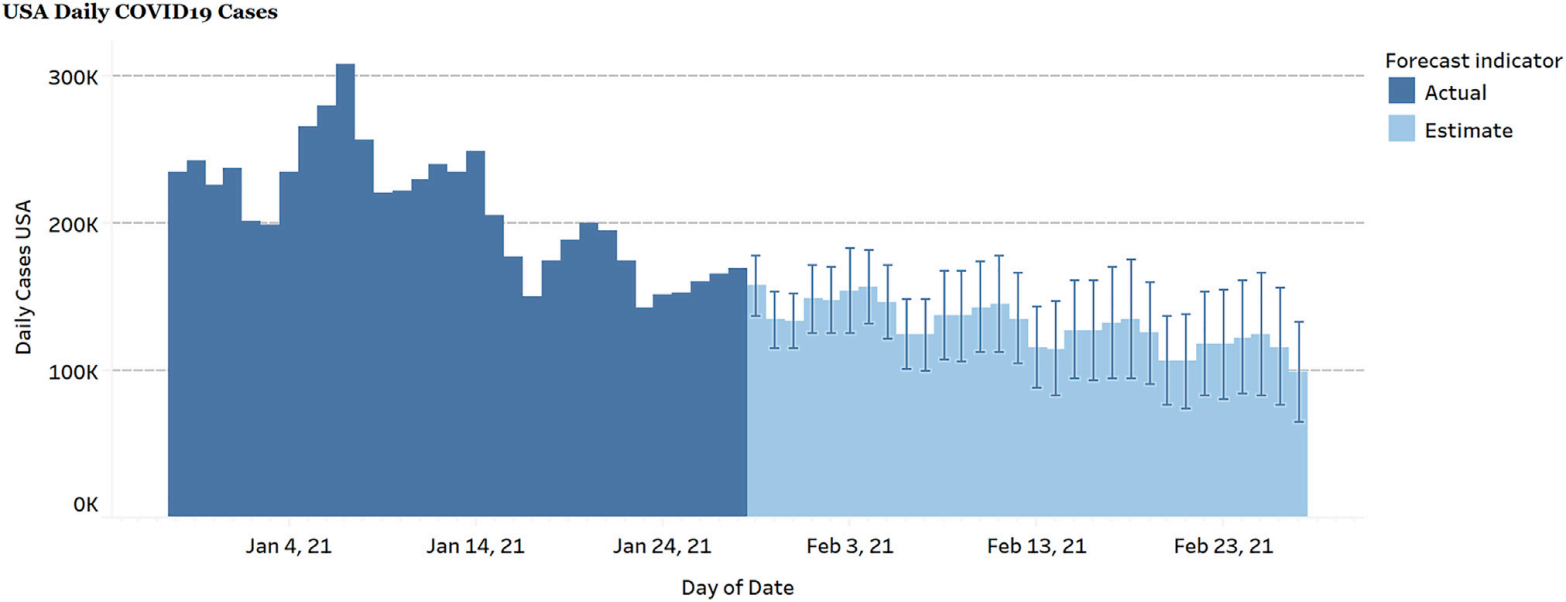
Forecasting daily COVID-19 infected cases in the United States

**Figure 7:**
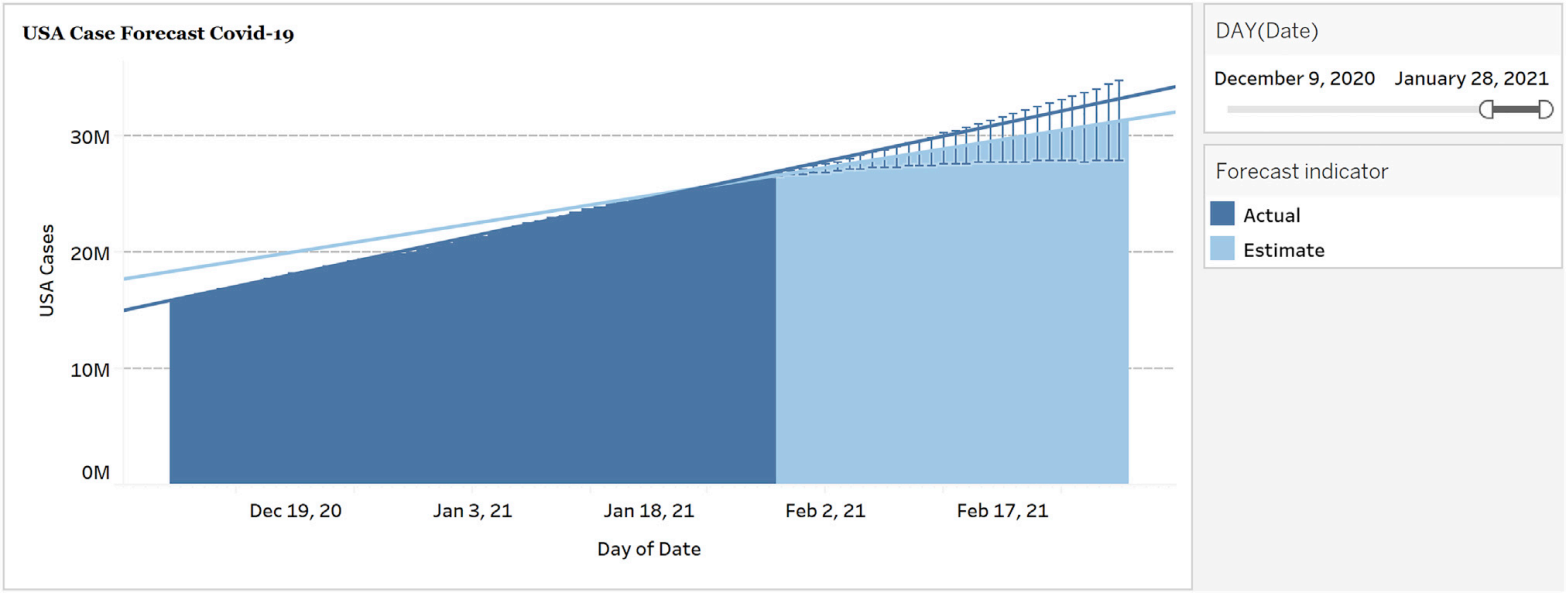
Forecasting total COVID-19 cases in the United States

**Figure 8:**
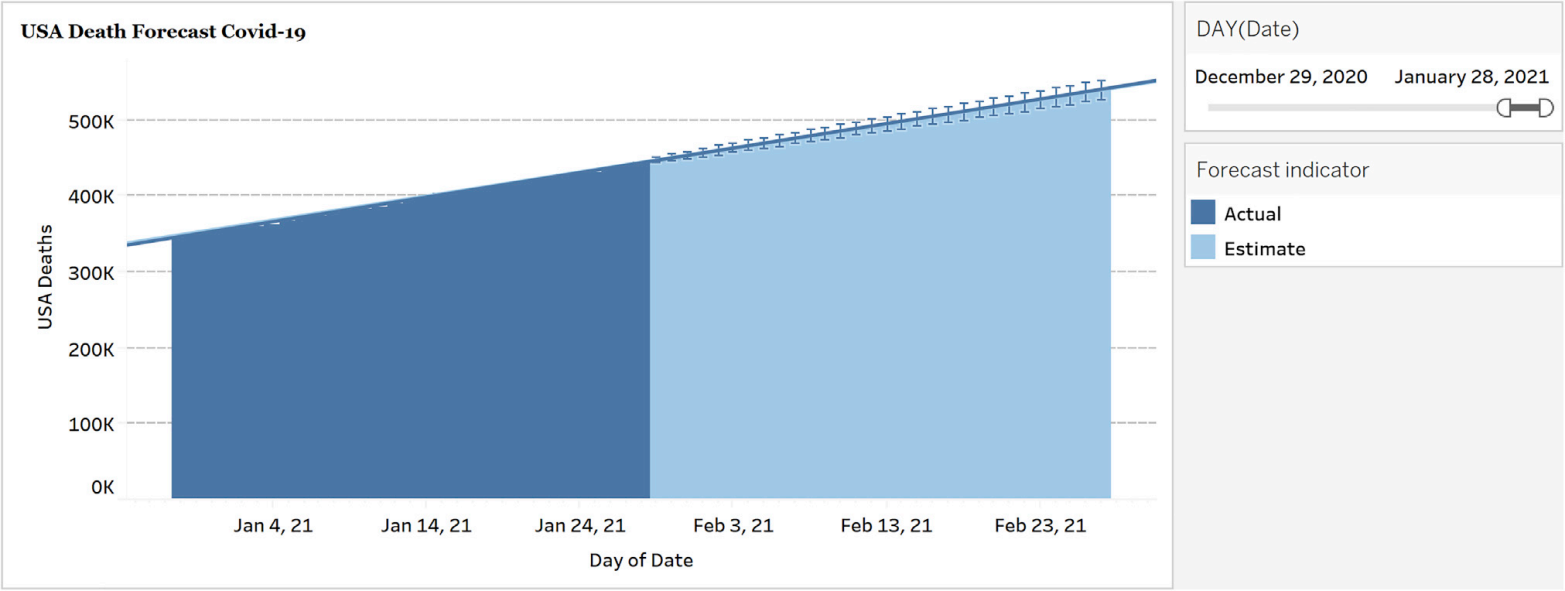
Forecasting COVID-19 death cases in the United States

After that, we simulated the COVID-19 cases for the United States. At first, we evaluated the daily infected COVID-19 cases in the United States (see Figure 6). We can observe that the daily new cases in the United States are decreasing in January 2021 and the model predicts the cases for February 2021 by maintaining a specific pattern. Further, in Figure 7, we present the total infected case forecast for the United States, and in Figure 8, we simulate the predicted results for US COVID-19 death cases in the United States. We can notice that we obtained small error bars for each predicted value, i.e., the difference between the predicted result and the actual value is pretty small. We used R-squared measurement, which is a statistical measure that represents how well the data fit the regression model, i.e., it indicates the closeness of data to the regression line. The definition of R squared can also be stated as the percentage of variation in a response or dependent variable that can be explained by an exploratory or independent variable(s):

(Equation 13) $$\text{R}-\text{squared}=\frac{\text{Explained variation}}{\text{Total variation}}.$$

The value of R squared is always between 0% and 100%. If the value of R squared equals 0%, this indicates that the model does not explain any variability of the output data around its mean, while a 100% R-squared value specifies that the model explains all the variability of the output data around its mean. The closer the R-squared value is to 100%, the better the fit of the model. Our prediction model has 99.3% R-squared value for world cases, 99.27% for world deaths, 99.1% for US COVID-19 cases, and 96.9% for US death cases. Similarly, we calculated the R-squared value considering the Dow Jones, S&P 500, and NASDAQ market prices and obtained results of 96.8%, 96.8%, and 93.5%, respectively. The R-squared values can be seen in our publicly available dashboard, which is published in Tableau.

**Figure 9:**
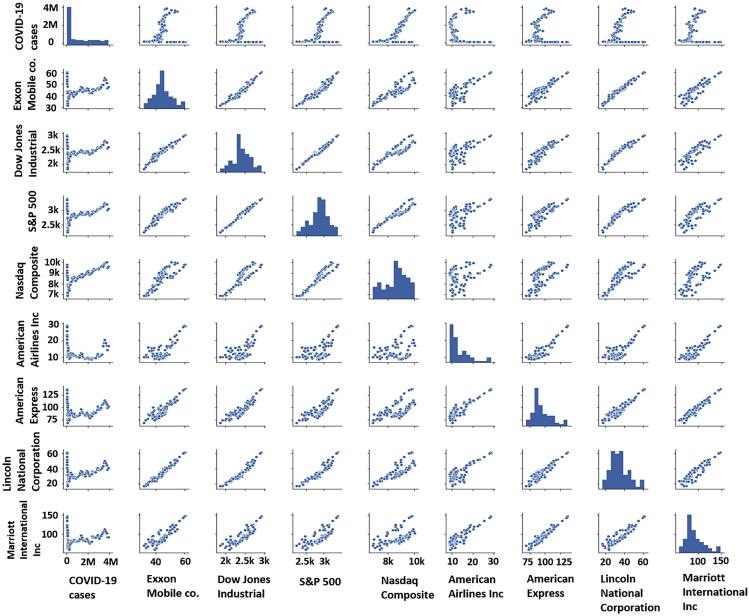
Visualization of correlation matrix of COVID-19 effects on top industries

After that, we simulated the correlation matrix that showed the impact of COVID-19 on top industries. In Figure 9, we present the visualization of the correlation matrix considering COVID-19 effects on those top industries.

**Figure 10:**
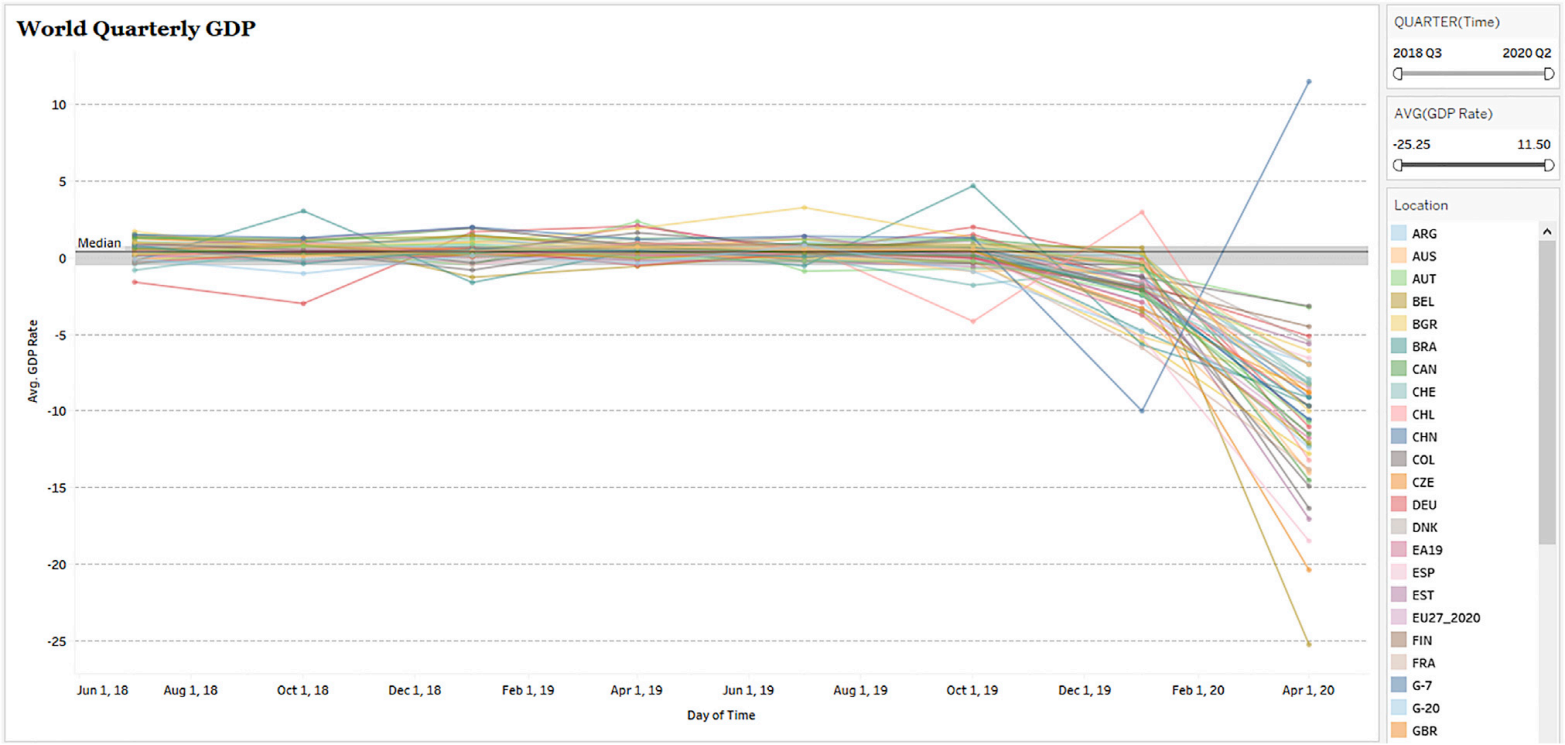
Effect of COVID-19 on worldwide GDP

**Figure 11:**
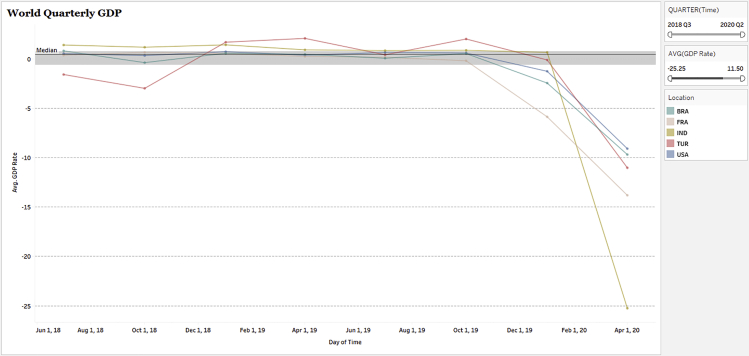
Effect of COVID-19 on GDP considering five different countries

Moreover, we analyzed the COVID-19 impact on GDP across countries of the world and discovered that almost all the countries' GDP went downward, except for China in the second quartile of 2020. In Figure 10, we present the average GDP of 40 countries, and for better understanding of the individual countries' GDP, in Figure 11, we present the average GDP rate of the five most-COVID-19-infected countries (i.e., United States, India, Brazil, France, Turkey), where the United States achieved the highest GDP rate in the second quartile among the five countries and India achieved the lowest GDP.

**Figure 12:**
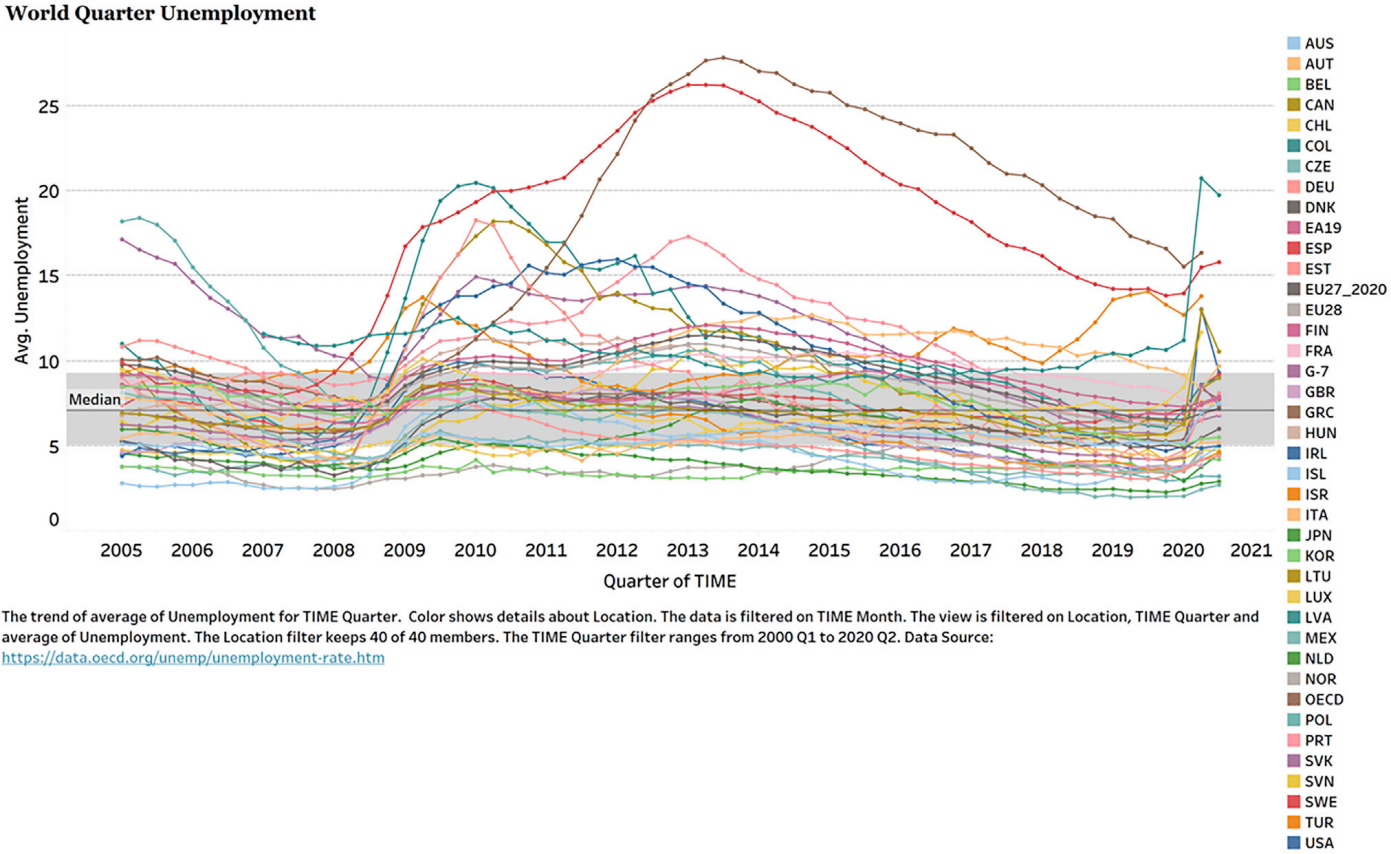
Effect of COVID-19 on worldwide unemployment

**Figure 13:**
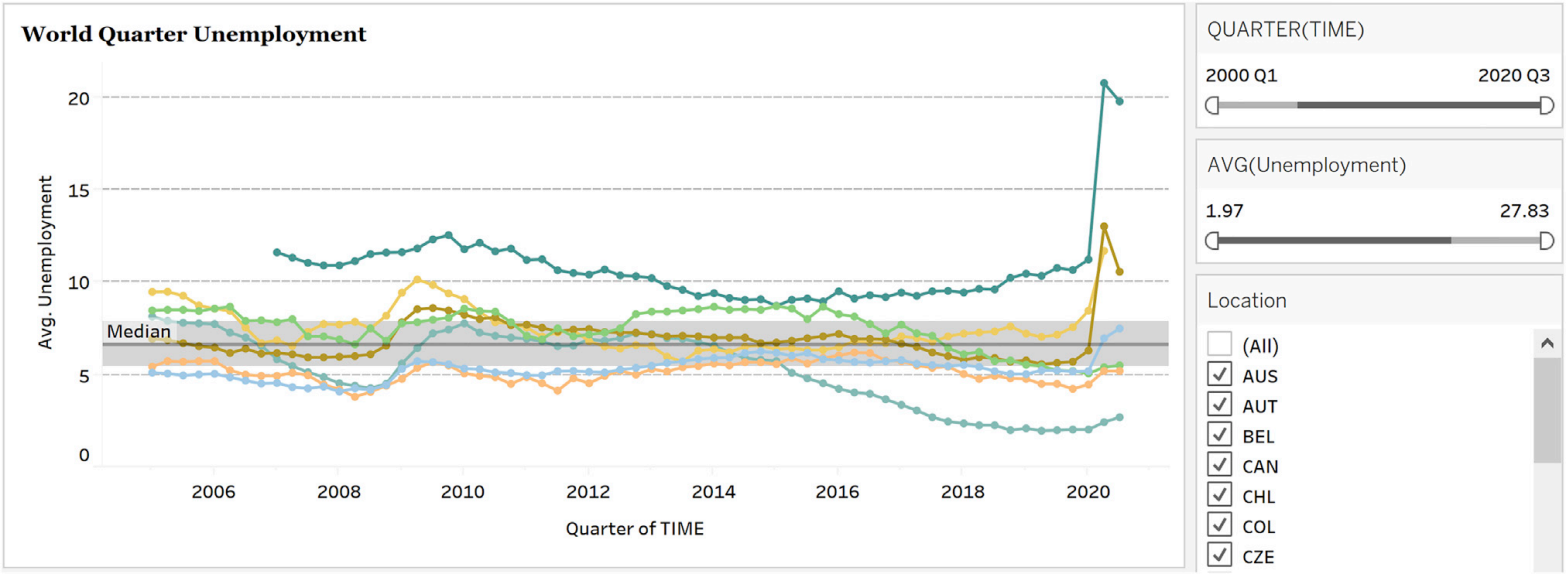
Effect of COVID-19 on unemployment considering seven different countries

Further, we analyzed the COVID-19 effects on worldwide unemployment. In Figure 12, we present COVID-19 effects on 40 countries, and for clear understanding the COVID-19 impact on unemployment rate, we present the average unemployment status and forecast of seven affected countries (see Figure 13). We predicted that the United States and Italy would have a maximum average unemployment rate in the first quartile of 2021. For better understanding of the graphs with details about the influence of COVID-19 on different countries’ GDP and unemployment status, we direct the reader to our publicly available dashboard.^1^

### Conclusion

In this paper, we proposed a COVID-19 prediction model that presents the impact of infectious disease and its shock waves on economy. We started developing and improving our model from the early stage of COVID-19 and thoroughly tested the actual events' performance. We achieved promising results in forecasting COVID-19 total cases, including the active and death cases around the world and in the United States. In addition, we revealed the economic impact experienced by top industries due to COVID-19 and presented a detailed graphical representation of such effects. By observing the upcoming impacts on those giant companies, people can understand the probable stock price shock waves in the coming days. Moreover, we analyzed the influence of COVID-19 on national GDP and unemployment rate and showed how GDP and unemployment population drastically changed following geographical dispersity. We evaluated our model quality through R-squared measurement and achieved significant model performance for prediction of COVID-19 cases as well as in forecasting its impact on GDP and unemployment rate. Our prediction schemes can be further applied to predict other countries' COVID-19 cases and reveal significant adverse consequences ahead of time. Our developed model would be effective in understanding the future demand of specific critical resources such as hospital equipment, e.g., oxygen mask, ventilator, hospital cabin, etc. In the future, we plan to consider the influence of infectious disease on human mobility and traveling behavior.

## Experimental procedures

### Resource availability

#### Lead contact

The lead contact for this paper is Dr. M. Hadi Amini, Assistant Professor, Knight Foundation School of Computing and Information Sciences, Florida International University, Miami, FL 33199, USA; email: moamini@fiu.edu.

#### Materials availability

Our developed data analytics dashboard for COVID-19 prediction is publicly available at the following link: https://public.tableau.com/profile/solid.lab#!/vizhome/DataAnalyticsforCOVID-19Prediction/Covid-19Dashboard.

#### Data and code availability

Our data and code are publicly available at the following GitHub repository: https://github.com/Imteaj10/Data-Analytics-for-COVID-19-Prediction-.
